# The role of research ethics committees in addressing optimism in sample size calculations: a meta-epidemiological study

**DOI:** 10.1186/s41073-025-00184-w

**Published:** 2025-12-12

**Authors:** Marieke S. Jansen, Rolf H. H. Groenwold, Olaf M. Dekkers

**Affiliations:** 1 Department of Clinical Epidemiology, Leiden University Medical Centre, Albinusdreef 2, Leiden, ZA 2333 the Netherlands; 2 Department of Biomedical Data Sciences, Leiden University Medical Centre, Leiden, the Netherlands; 3 Department of Endocrinology and Metabolic Disorders, Leiden University Medical Centre, Leiden, the Netherlands; 4 Department of Clinical Epidemiology, Aarhus University and Aarhus University Hospital, Aarhus, Denmark

**Keywords:** Effect size, Target difference, Sample size calculation, Optimism bias, Clinical trials, Research ethics committee, Ethics approval

## Abstract

**Background:**

Sample size calculations are critical in clinical trial design, yet hypothesised effect sizes are often overly optimistic, leading to underpowered studies. Research ethics committees (RECs) assess trial protocols, including sample size justification, but their role in mitigating optimism bias in sample size calculations is not well studied.

**Methods:**

We descriptively analysed 50 clinical trial protocols approved by a Dutch REC (2015–2018) with available primary outcome results. We examined REC comments on sample size calculations, protocol modifications during ethics review and amendments, and discrepancies between target and observed effect sizes. For comparability, effect sizes were standardised.

**Results:**

Nine (18%) trials received REC comments on sample size calculations, mainly addressing calculation errors (*n* = 5), missing parameters (*n* = 2), or other methodological considerations (*n* = 3), with only three comments (6%) requesting effect size justification. Seven (14%) trials modified their sample size calculation during ethics review, mostly in response to REC comments, and 10 (20%) trials made modifications in amendments. In total, 40 (80%) trials overestimated their target effect size. Across all trials, the target effect size was overestimated by a median of 0.22 [IQR: 0.03 – 0.41]. Changes during ethics review led to less overestimation for only one trial, which reflected the correction of a calculation error rather than a reassessment of assumptions.

**Conclusions:**

Optimism in sample size calculations is common, but the influence of REC feedback on reducing overestimation appears limited. As this was a small, descriptive study from a single Dutch REC in 2015–2018, findings may not generalise to other settings or more recent practice. Future research should validate these findings and may help identify characteristics associated with overestimation, supporting RECs in recognising trials at risk of being underpowered.

**Supplementary Information:**

The online version contains supplementary material available at 10.1186/s41073-025-00184-w.

## Background

Sample size calculations play a fundamental role in the design of clinical trials and interpretation of their results. They help ensure that a study has sufficient probability (i.e., statistical power) to detect a particular treatment effect, should it exist [1]. These calculations rely on several parameters, some of which are relatively fixed, such as type I and type II error probabilities. Others, particularly the hypothesised effect size or target difference, can be determined with considerable flexibility. The hypothesised effect size has a large impact on the required sample size, and should be clinically meaningful and plausible [2].

Prior research suggests that hypothesised effect sizes in sample size calculations of clinical trials are frequently overestimated [3–5]. A recent study of cardiovascular trials published in high impact journals found that approximately 82% of the trials had an hypothesised effect size larger than those eventually observed, and approximately 61% overestimated event rates of the control group [6]. The use of overly optimistic parameters leads to underpowered trials, increasing the chance to miss clinically relevant effects, while effects that are detected are likely exaggerated [7, 8], contributing to inconclusive and potentially misleading results.

Research ethics committees (RECs) evaluate clinical trial protocols to ensure ethical standards are met, which includes an evaluation of methodological quality and the required sample size. We sought to examine how often trial protocols receive comments on sample size calculations during ethics review, for example regarding the justification of chosen parameters. We also aimed to examine how often and why calculations are changed during initial ethics review and later amendments. Subsequently, we aimed to explore the degree of potential optimism in hypothesised effect sizes for trials with and without REC comments, modifications, and varying trial characteristics.

## Methods

For reporting our study, we followed an adapted version of the PRISMA guideline for reporting meta-epidemiological studies, using items where applicable [9].

### Study design and sample

This study utilised data from a previous study, which involved a cohort of trials investigating healthcare interventions, approved in the period 2015–2018 by a Dutch REC [10]. This dataset included information on trial completion status, trial characteristics, and elements of the ethics review process (e.g., review durations and REC comments), extracted from archived trial protocols, correspondence between the REC and investigators, and related study documents. Detailed methods have been described previously [10], and its protocol is available from https://osf.io/ucn3j. The present analysis does not have a separate protocol registered. Trials from this cohort were included in the present study if: (1) they involved a two-arm parallel design with a superiority hypothesis, and (2) results of the primary outcome were available in a peer-reviewed publication, trial record or results report submitted to the REC. Trial designs other than a simple two-arm parallel trial (e.g., single arm, crossover, group-sequential, adaptive, cluster randomised trials, etc.) were thus not included. Trials were furthermore excluded if the achieved sample size was < 0.4 of the target sample size (similar to Zakeri et al.) [5], or if not enough information from the protocol and publication was available to calculate a standardised target effect size and standardised observed effect size, respectively. Assessment of eligibility was performed by one author (M.J.). Details regarding the structure and function of the REC under study are outlined in the Supplementary Material.

### Data collection

From the dossiers of the REC and related published research articles, one author (M.J.) extracted information regarding: (1) characteristics of sample size calculations in protocols and subsequent publications, including potential modifications, (2) sample size comments raised during ethics review, and (3) results of the primary outcome.

#### Characteristics of sample size calculations and potential modifications

Data on sample size parameters were extracted from trial protocols (initial and amended) and subsequent publications, if available. Extracted parameters included type I error probability (alpha), type II error probability (beta, i.e., 1—power), one- or two-sided hypothesis test, allocation ratio, attrition rate, and resulting sample size, which were used to calculate standardised target effect sizes (see Supplementary Material). Additionally, the method used to elicit the target difference was extracted and classified according to the DELTA2 guideline [2]. Reasons for potential sample size changes were extracted from correspondence between the REC and investigators. We considered the sample size calculation as completely reported if all components to be able to recalculate the sample size were available (e.g., type I and type II error probabilities, target difference, variability or event rate in the control arm, hypothesis test side).

#### Sample size comments raised during the ethics review

REC comments addressing sample size calculations were classified into five categories: (1) absence of a sample size calculation, (2) unclear or missing parameters, (3) request for parameter justification, (4) potential calculation errors, and (5) other (e.g., consideration of multiple testing, multiple primary outcomes).

#### Primary outcome results

Peer-reviewed results publications were identified via PubMed and Google Scholar searches by entering trial registration numbers, local study identifiers, REC identifiers, study titles, intervention names, and principal investigator names. We additionally checked trial records for any linked publications. A list of trial registration numbers of the included clinical trials are available in the Supplementary Material. Publications were only considered if they included the results of the primary outcome (i.e., the outcome that the sample size calculation of the approved protocol was based on). If no publication was found, results were extracted from the trial record or results report submitted to the REC, if available. For the primary outcome, the observed effect size, standard error and p-value were extracted to calculate standardised observed effect sizes. Additionally, we retrieved significance status (per each trial’s predefined significance level), achieved sample sizes and attrition numbers.

### Statistical analysis

Standardised effect sizes were calculated using similar methods as Rothwell et al. [11]. Instructions and formulae used are detailed in the Supplementary Material. For 4 (8%) protocols, the type I error probability (or statistical significance level), and for 20 (40%) protocols the hypothesis test side was not explicitly reported in the protocol. In order to still calculate standardised target effect sizes for these trials, we assumed these parameters to be set at 5% and two-sided, respectively. These assumptions were checked and matched with corresponding results publications for each trial.

Binary variables were summarised as frequencies and proportions, while continuous variables were reported as medians with interquartile ranges. Potential optimism of the target effect size was quantified as the discrepancy between the standardised target effect size of the final approved protocol and the standardised observed effect size (standardised target effect size – standardised observed effect size). The relative discrepancy was quantified as the discrepancy divided by the standardised target effect size. No hypothesis testing was applied.

All analyses were performed using R (version 4.2.1).

## Results

### Description of included trials

**Fig. 1:**
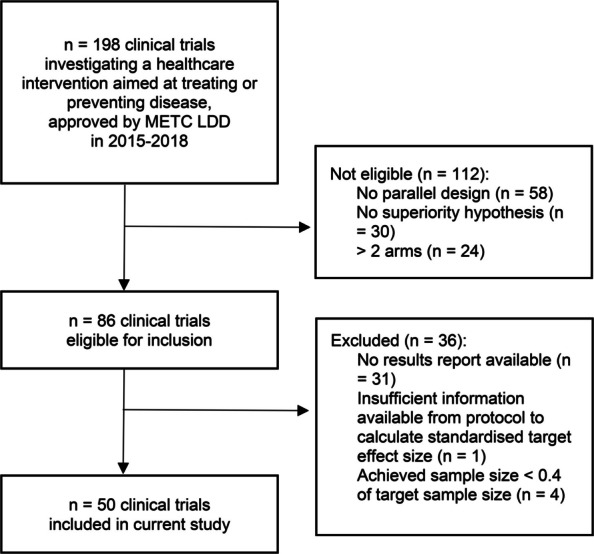
Flowchart of study inclusion

A total of 86 trials were eligible for inclusion. Of these, 36 trials were excluded due to a lack of available results reports that contained the primary outcome of interest (*n* = 31), insufficient information available from the protocol to calculate a standardised target effect size (*n* = 1), or an achieved sample size of < 0.4 of the target sample size (*n* = 4). This resulted in 50 clinical trials being included in the current study (Fig. 1). Characteristics of the these trials are outlined in Table 1. All trials were completed (*n* = 39) or terminated early (*n* = 9) between 2016–2024, except for two trials for which long term follow-up was still ongoing. Results were extracted from peer-reviewed publications or preprints (*n* = 44), results reports submitted to REC (*n* = 5) or the corresponding trial record (*n* = 1). Exactly twenty-five (50%) out of 50 trials observed a positive (statistically significant) result. Stratified by sponsor, this included 10 (67%) out of 15 industry trials, and 15 (43%) out of 35 investigator-initiated trials.

**Table 1:** Trial characteristics of the included trials

Variable	*n* = 50	%
Sponsor		
Investigator	35	70
Industry	15	30
Subsidising party (other than the sponsor)		
None	24	48
Industry^a^	13	26
Other^b^	13	26
Centres		
Single centre	17	34
Multicentre	33	66
Medical field		
Internal medicine	9	18
Oncology	7	14
Neurology & neurosurgery	8	16
Psychology & psychiatry	6	12
Other^c^	20	40
Intervention		
Drug^d^	31	62
Device	3	6
Behavioural & digital health innovations	9	18
Other^e^	7	14
Phase		
1	0	0
2	10	20
3	11	22
4	3	6
Other^f^	7	14
Not applicable	19	38
Comparator		
Active	24	48
Placebo	17	34
No intervention	9	18
Allocation		
Randomised	50	100
Nonrandomised	0	0
Masking		
Open	20	40
Blinded	30	60
Target sample size (median [IQR])	115	[80–200]
Achieved sample size (median [IQR])	111.5	[66.25–203]

^**a**^Industry category also includes trials that had a combination of industry and other subsidising parties

^b^Other includes any non-commercial subsidising party such as foundations, non-profit organisations, etc.

^c^Other includes anaesthetics, emergency medicine, general surgery, immunology, paediatrics, public health, rehabilitation medicine, rheumatology, urology

^d^Drug intervention also includes biologicals

^e^Other includes dietary interventions, surgical interventions and other interventions that did not fit into any of the listed categories

^f^Medicinal product trials that did not fit in phase 1–4 and were classified as ‘other phase’ by the principal investigator

### General characteristics of sample size calculations in protocols

Sample size calculations of final approved protocols were complete in 25 (50%) out of 50 trials, while 18 (41%) out of 44 were completely reported in respective peer-reviewed publications (Table 2). Overall, completeness did not appear to improve between first submitted and final approved protocols (the only changes were a slight decrease in missing variability from 20 to 18%, while missingness of the target effect size increased from 4 to 12%). Parameters that were most often missing included specification whether hypothesis testing was one- or two-sided (*n* = 20, 40%), followed by the variability or event rate of the control group (*n* = 9, 18%). The most common method to elicit the target effect size were literature reviews (16 (32%) trials in combination with mixed methods) (Table 3). In 23 (46%) protocols, information regarding how the target effect size was substantiated was missing.

**Table 2:** Completeness of sample size sections across protocol versions and the publication (if available)

	First submitted protocol		Final approved protocol^b^		Publication	
	*n* = 50	%	*n* = 50	%	*n* = 44	%
Complete	25	50	25	50	18	40.9
Calculation absent	0	0	0	0	1	2.3
Missing parameters						
Type I error (alpha)	4	8	4	8	6	13.6
Type II error (beta)	0	0	0	0	1	2.3
One—or two-sided hypothesis test	20	40	20	40	19	43.2
Target effect size	2	4	6	12	2	4.5
Variability or event rate control arm	10	20	9	18	9	20.5
Other^a^	4	8	4	8	6	13.6

^a^E.g., correlation between repeated measurements if applicable, number of required events in time-to-event analyses, number of required participants

^b^Most recent protocol version (e.g., either after initial ethics review, or after amendments if applicable)

**Table 3:** Method of elicitation of the target effect size and variability or event rate in the control group

	Target effect size		Variability	
Method of elicitation^a^	*n* = 50	%	*n* = 50	%
Literature review	10	20	15	30
Pilot study^b^	3	6	11	22
Opinion-seeking	2	4	0	0
Standardised effect size	4	8	4	8
Mixed methods^c^	6	12	6	12
Other^d^	2	4	0	0
Not mentioned	23	46	14	28

^a^As specified in the final approved protocol

^b^Phase 1–2 studies, unpublished internal data, and observational variants of the trial conducted by the authors (e.g., similar to control arm) were also considered as pilot studies

^c^Mixed methods consisted of literature reviews in combination with opinion-seeking, standardised effect size and/or pilot study

^d^Other included all other justifications (e.g., phrases including “We consider a difference of […] relevant.”, and “Minimal clinically important difference of […]”, without further reference.)

### Comments raised regarding the sample size calculation during ethics review

Nine (18%) out of 50 trials received REC comments regarding the sample size calculation during ethics review (*n* = 7 received one comment, *n* = 1 received 2 comments, and *n* = 1 received 4 comments). Six (67%) of these 9 trials did not have a complete sample size calculation. The core issues raised during ethics review included potential errors in the calculation (i = 5), request for clarification of unclear or missing parameters (*n* = 2), request for substantiation of chosen parameters (*n* = 3), and other (e.g., accounting for multiple testing in case of multiple outcomes or interim analyses, *n* = 3). Of the 41 (82%) protocols without REC comments on the sample size calculation, approximately half showed incomplete reporting and lacked justification for the target effect size. Full details are provided in Supplementary Table S1.

### Sample size calculation modifications during ethics review and amendments

Seven (14%) out of 50 trials modified the sample size calculation section of the protocol during ethics review (*n* = 6 had 1 modification, and *n* = 1 had 3 modifications). Ten (20%) out of 50 trials modified the sample size calculation in amendments, after already having started (*n* = 9 had 1 modification, and *n* = 1 had 2 modifications). Modifications during ethics review were mostly in response to REC comments (8 out of 9 modifications), while modifications during amendments were all initiated by investigators (Table 4). Modifications resulted both in increases and decreases of the total required sample size, or no change at all. Additionally, modifications during ethics review resulted into a decrease of the target effect size for only 2 trials, of which only 1 decrease appeared relevant in relation to the eventual observed effect size after correcting for an error (trial #5, Table 4). Modification reasons and additional details are outlined in Table 4.

**Table 4:** Sample size modifications characteristics

Trial	Version change	Target ES (before → after)	Total sample size change	Reason	Induced by	Observed ES
*Modifications during ethics review*						
1	1 st → 2nd	1.03 → 1.03	0%	Clarification of parameters	REC	0.50
2	1 st → 2nd	1.03 → 1.03	0%	Clarification of parameters	REC	0.69
3	1 st → 2nd	0.83 → 0.59	0%	Correction error	REC	0.83
4	1 st → 2nd	0.70 → 0.61	+ 30%	Correction error, clarification and substantiation of chosen parameters	REC	0.77
4	2nd → 3rd	0.61 → 0.81	−42%	Correction error	REC	0.77
4	3rd → 4th	0.81 → 0.75	+ 14%	Correction error	REC	0.77
5	1 st → 2nd	0.81 → 0.70	+ 50%	Correction error	REC	0.58
6	1 st → 2nd	0.39 → 0.39	−18%	Correction error	REC	0.37
7	1 st → 2nd	0.33 → 0.44	−42%	Change in primary outcome measure and chosen parameters	Investigator	0.42
*Modifications during amendments*						
1	1 st → 2nd	0.42 → 0.42	+ 15%	Adjustment for dropout	Investigator	0.03
2	1 st → 2nd	0.72 → 0.72	−11%	Adjustment for dropout	Investigator	0.22
3	1 st → 2nd	0.60 → 0.51	+ 43%	Decrease of target effect size based on external evidence, increase power for secondary outcomes, adjustment for dropout	Investigator	0.47
4	1 st → 2nd	0.40 → 0.43	+ 13%	Increase of target effect size based on external evidence, increase power	Investigator	0.54
5	1 st → 2nd	0.50 → 0.68	−43%	Preplanned interim analysis for sample size recalculation	Investigator	0.44
6	1 st → 2nd	0.77 → 0.57	+ 60%	Preplanned interim analysis for sample size recalculation	Investigator	0.55
6	2nd → 3rd	0.57 → 0.61	+ 12%	Change of primary outcome and increase of power	Investigator	0.55
7	1 st → 2nd	0.95 → 0.86	+ 6%	Change of primary outcome	Investigator	0.07
8	1 st → 2nd	0.40 → 0.55	−47%	Change of primary outcome	Investigator	1.02
9	1 st → 2nd	0.51 → 0.51	−33%	Increase of alpha and power, change from confirmatory to exploratory aim	Investigator	0.45
10	1 st → 2nd	0.50 → 0.50	−52%	Introduction of crossover arm to account for recruitment failure	Investigator	0.06

### Potential optimism in sample size calculations

**Fig. 2:**
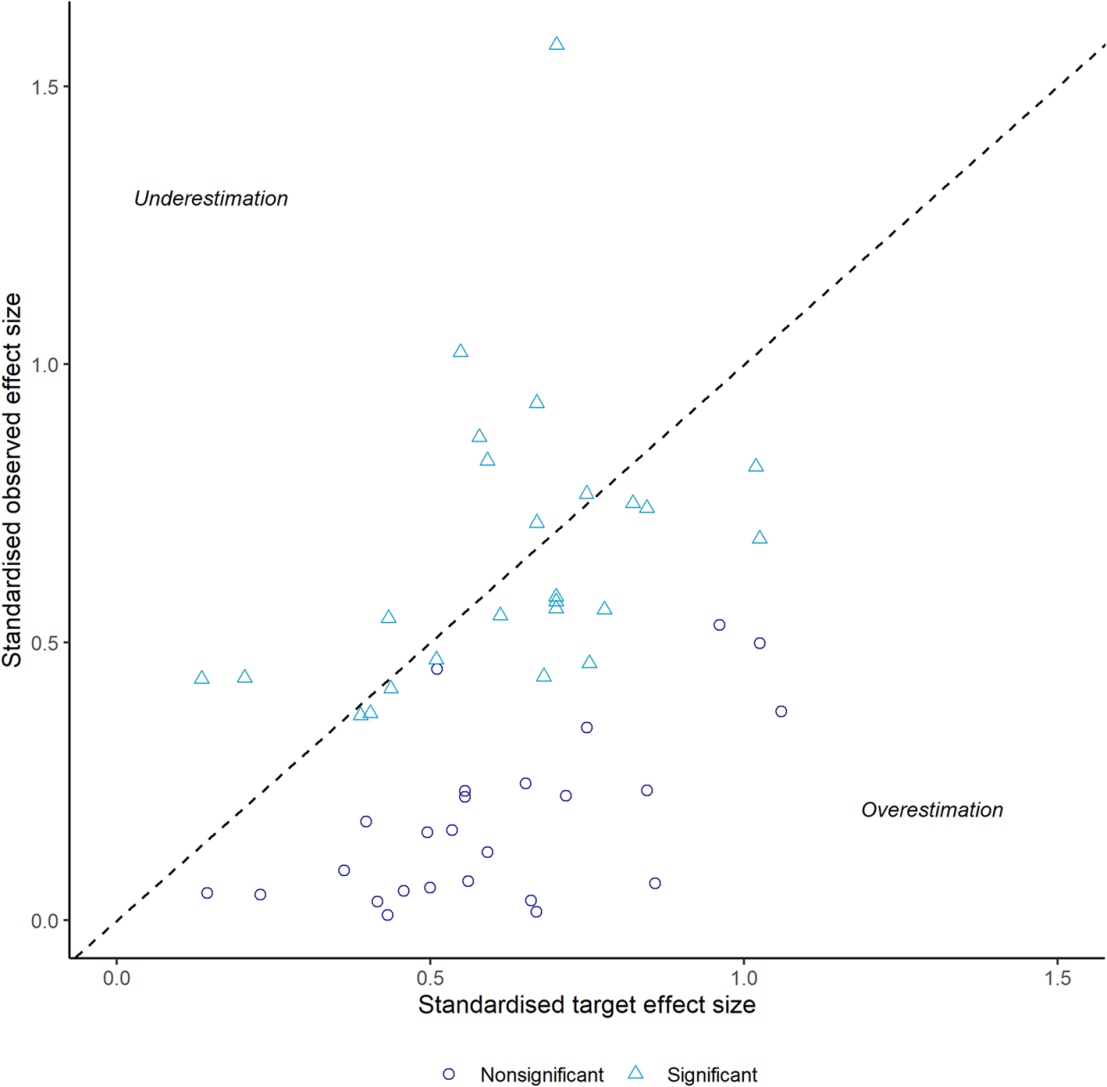
Scatterplot of standardised target effect sizes and standardised observed effect sizes

The median standardised target effect size of final approved protocols was 0.60 [IQR: 0.47–0.74] and the median standardised observed effect size was 0.43 [IQR: 0.16–0.57]. In total, 40 (80%) trials overestimated the target effect size, and 10 (20%) trials underestimated the target effect size (Fig. 2). Median overestimation of the target effect size across all trials was 0.22 [IQR: 0.03–0.41]. In relative terms, the median overestimation was 59% [IQR: 9% – 226%].

**Fig. 3:**
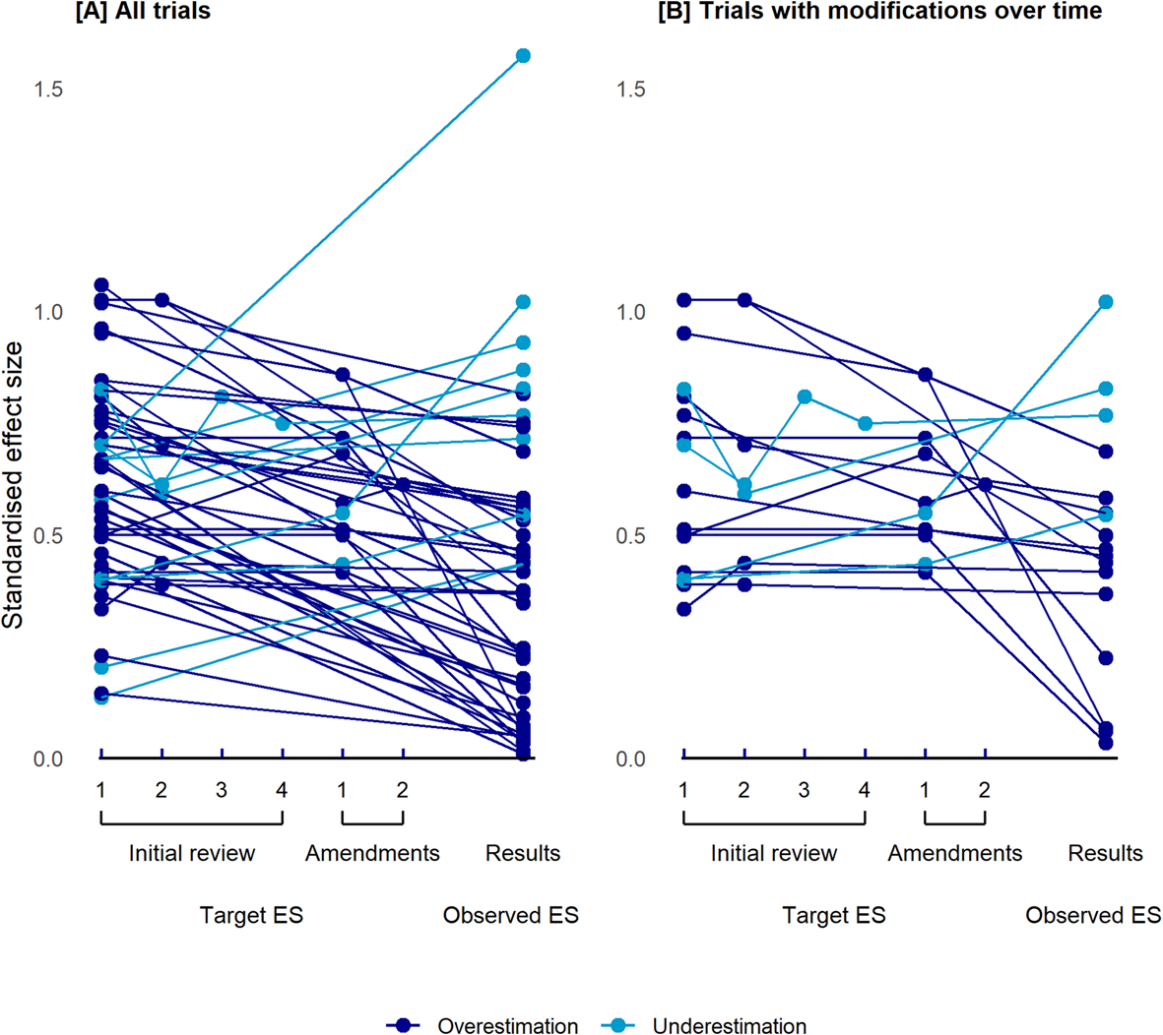
Individual trajectories of the standardised target effect size across protocol versions and the standardised observed effect size for each trial. [A] All trials (*n* = 50). [B] Trials with modifications of the sample size calculation section during initial ethics review and/or amendments (*n* = 17). All effect sizes are standardised. ES = Effect size

Figure 3A illustrates individual trajectories of the standardised target effect size across protocol versions during ethics review and amendments, as well as the standardised observed effect size, for each trial. Modification of the sample size calculation section during ethics review (*n* = 7) resulted in a small decrease of the standardised target effect size overall (first submitted protocol, median: 0.81 [IQR: 0.54–0.93]; first approved protocol, median: 0.70 [IQR: 0.51–0.89]). Two trials contributed to this decrease, while for five trials the standardised target effect size remained the same or increased slightly (Table 4 and Fig. 3B). Modifications of the sample size calculation during amendments resulted in a negligible increase overall (first approved protocol, median: 0.51 [IQR: 0.44–0.69], final approved protocol after amendments, median: 0.53 [IQR: 0.50–0.66])(Table 4, Fig. 3B).

**Fig. 4:**
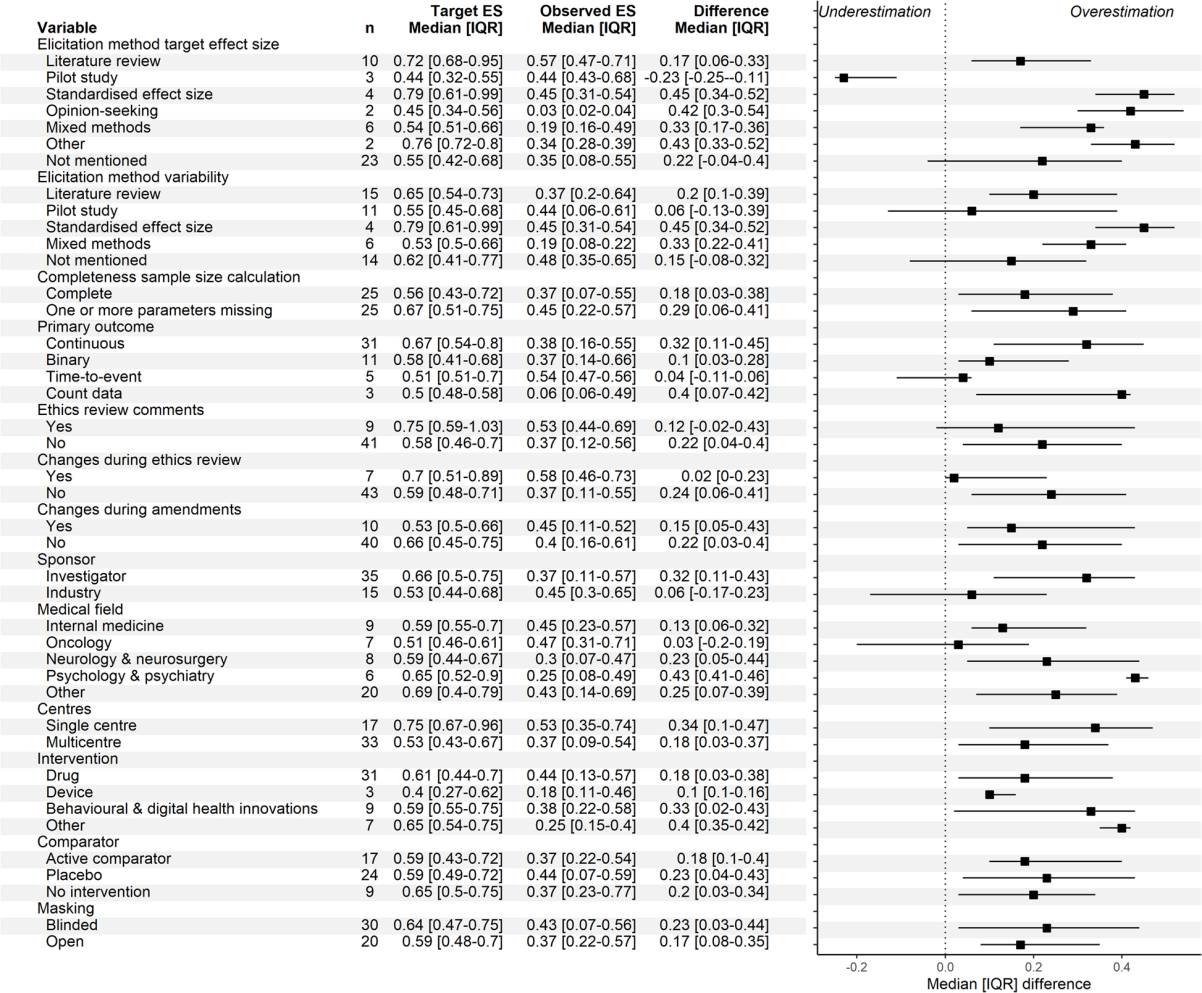
Potential optimism of the target effect size, stratified by sample size calculation characteristics, REC comments, sample size modifications, and trial characteristics. All effect sizes are standardised. The target effect size is from the final approved protocol. The difference between the target effect size and observed effect size is calculated as target effect size – observed effect size. Due to few observations for some categories in combination with skewed distributions, over- or underestimation of the target effect size can be present even though medians of the standardised target and observed effect size are similar. ES = Effect size; IQR = Interquartile range

Potential over – or underestimation of the target effect size stratified by sample size characteristics, REC comments, modifications and trial characteristics are outlined in Fig. 4. For certain subgroups the median difference between the target and observed effect size was small or even negative (i.e., indicating underestimation), including trials that based their target effect size and/or variability off pilot data, trials with time-to-event outcomes, those with sample size calculation changes during ethics review or amendments, industry sponsored trials, and trials conducted within the field of oncology. Subgroups that had large overestimations included trials that used the standardised effect size or opinion-seeking method to inform sample size parameters, were conducted in the field of psychology and psychiatry, and investigated other interventions.

When stratified by study outcome, completeness of reporting and justification of sample size parameters did not differ appreciably between positive (significant) and negative (non-significant) trials. Similarly, target effect sizes were not larger in magnitude among negative trials. As visible in Fig. 2, overestimation was more apparent in negative trials. Further details are provided in Supplementary Table S2.

## Discussion

We sought to examine how often trial protocols receive comments on sample size calculations during ethics review, and how often and why calculations are changed during initial ethics review and later amendments. Subsequently, we aimed to explore the degree of potential optimism in hypothesised effect sizes for trials with and without REC comments, modifications, and varying trial characteristics.

About 1 in 5 trials in our sample received comments during ethics review regarding the sample size calculation. Comments regarding the justification of chosen parameters were only raised for 3 (6%) trials. Instead, the majority of the comments addressed technical aspects of the calculation itself and clarity, and resulted in a small decrease of the standardised target effect overall. Additionally, 1 in 5 trials changed their sample size calculation during amendments, with either small increases or decreases of the standardised target effect size, ultimately resulting in a negligible difference of the target effect size overall. Reasons for sample size modifications during amendments varied (e.g., from spontaneous protocol adjustments and incorporation of observed dropout rates to preplanned interim sample size recalculations).

Overly optimistic assumptions in sample size calculations lead to underpowered trials that are more likely to yield inconclusive and potentially misleading results. As expected, overestimation was most apparent among negative trials, reflecting the inherent relation between optimistic effect assumptions and the likelihood of inconclusive findings. In our sample, 80% of the trials overestimated the standardised target effect size (a composite measure of the hypothesised effect and variability). This finding is similar to what has been reported for (non-standardised) target effect sizes by others, with proportions of approximately 82% [6], 83% [3], and 90% [5]. The proportion of trials with significant results was relatively high in our sample (50%) compared to other studies (43% [6], 27% [3], 22% [5]). Particularly industry sponsored trials had a high proportion of positive trials (67% vs. 43% for investigator-initiated trials). The high number of positive trials in our study might at least partly be explained by the selection mechanism of our study in combination with relatively short follow-up times since trial completion. In contrast to other studies selecting publications during a specific time period, we followed a cohort of trials from ethics approval onwards, and subsequently selected on the availability of primary outcome results at one timepoint. About half of the trials we could not include due to missing results, only completed their trial recently (< 2.5 years). Trials with negative results take a longer time to publish (also known as “time-lag bias”) [12]. Consequently, we may have particularly missed negative trials, leading to higher proportions of significant results (and likely less overestimation) in our specific sample.

Similar to others [13, 14], we found 50% of trials not reporting all parameters used for the sample size calculation, which appeared worse for respective publications, even though reporting guidelines for protocols and publications (e.g., SPIRIT, ICH, CONSORT) have been available for a substantial time (e.g., up to two decades for ICH and CONSORT at time of ethics approval for trials in our sample) [15–17]. Furthermore, only 54% justified their chosen target effect size, similar to 43% of UK study protocols reported by Clark et al. [14]. We did find a larger proportion of trials that justified the population variability or event rate in the control group in comparison to what has been reported previously (72% vs. 48%) [14]. While the REC addressed such shortcomings for some protocols, reporting was frequently incomplete and justification of the target effect size was often lacking in protocols without any comments, suggesting that some of these issues may have been overlooked during ethics review. Potential overestimation of the standardised target effect size varied across subgroups. While sample size comments and subsequent modifications during ethics review seemed to result in less overestimation overall, this was effect was driven by a single trial in which an error was corrected following REC feedback. Most other trials that revised their sample size section during ethics review had only a minor decrease, increase, no change to the target effect size at all, and/or did not result in less overestimation (e.g., the original target effect size was close to the eventual observed effect size). Thus, although REC comments occasionally prompted corrections, or even requested justifications for chosen parameters for 3 protocols, they seldom lead to meaningful reconsideration of underlying assumptions, and their overall impact on reducing overestimation of effect sizes appeared limited in our sample.

Notably, overestimation was small for industry sponsored trials, and appeared worse for investigator initiated trials. A study of cardiovascular trials published in high impact journals did not find an association between sponsorship and accuracy of the target effect size [6]. This difference might be explained by a different selection of trials (i.e., trial protocols submitted to REC vs. trials published in high impact journals). Furthermore, overestimation varied across other subgroups, although most of these subgroups are likely correlated with one and another, as well as to sponsorship. For example, minimal or no overestimation was observed in trials using pilot data to inform the target effect size, time-event-outcomes, and oncology trials, which are likely also more often sponsored by industry. In contrast, substantial overestimation was observed in those using standardised effect sizes or expert opinion to inform sample size parameters, as well as in psychology and psychiatry trials (which commonly use standardised effect sizes and are typically investigator initiated).

Based on the current findings, it could be worthwhile to perform a larger scale study of trial protocols submitted to RECs and examine associations between overestimation of the target effect size and REC comments, modifications of the sample size over time and other characteristics. Although optimism of the target effect size was common, comments by REC reviewers regarding justification of the target effect size were rare. Likely, it is challenging for reviewers to gauge whether a hypothesised effect is plausible, and depends on many factors, such as the medical field, intervention, comparator and outcome of interest. Evaluating the plausibility of an assumed effect size is not purely a statistical judgment, as it requires knowledge of the topic under investigation. Accordingly, RECs might reconsider whether the assessment of sample size calculations should rest solely with the statistician. Identification of potential “red flags” might help REC reviewers to pinpoint trial protocols with a high risk of overestimation. Moreover, few resources currently provide RECs with concrete methodological guidance for evaluating sample size calculations. A recent scoping review of resources available to ethics committees identified over 200 templates, checklists, and guidelines, but noted a gap in support for methodological aspects, including sample size calculations [18]. This aligns with our local experience: although reviewer templates include sections for different reviewer roles (e.g., methodologists, jurists, ethicists), they tend to remain general and do not probe into sample size specifics beyond asking reviewers to “check” the calculation. Developing more explicit guidance (e.g., requiring applicants to provide a clear justification for both the anticipated effect size and the assumed variability) could help improve the quality and consistency of REC assessments, as well as ultimately the quality of clinical trials.

## Limitations

As observed effect sizes are an estimate of the true effect and subject to chance, it is possible that the proportion of overestimation of hypothesised effect sizes against true effects could differ from the 80% we have observed. Inevitably, we’ve only looked at trials that had results available for the primary outcome, although we were able to include results reports submitted to the REC if available (which most other studies do not have access to). However, only about half of the trials were compliant with the REC and submitted their results reports, which resulted in two additional results that otherwise would not have been available from public resources (e.g., peer-reviewed publication or trial record). As mentioned earlier, we may also have particularly missed trials with negative results due to time-lag bias in combination with our limited follow-up time, which may have led to higher observed proportions of significant results and lower estimates of overestimation. Furthermore, because REC dossiers generally lack information on the broader research team, we could not determine whether trained biostatistical expertise was involved in the design of the included studies. Additionally, we only investigated ethics review comments of one Dutch academic REC (which included two methodologists at the time) and only covered the period 2015–2018. While our findings may be generalisable to other Dutch academic RECs, they may not reflect commercial RECs or the CCMO, which evaluate different types of trials under varying review processes. Changes in REC composition and regulatory frameworks since the study period (e.g., CTIS implementation and evolving European regulations) may also limit the applicability of our results to more recent practice. Finally, our study was small and descriptive. Replication on a larger scale, preferably with longer follow-up time since trial completion, is necessary to validate our results.

## Conclusion

Optimism in sample size calculations is common, with the majority of clinical trials overestimating the target effect size. Our findings suggest that sample size calculation comments raised during ethics review mainly address calculation errors and other technical aspects, while comments regarding the justification of hypothesised effect sizes are relatively rare. Notably, a relevant reduction of the target effect size occurred in only one trial, which reflected the correction of a calculation error rather than a reconsideration of underlying assumptions. Overall, the influence of REC review on reducing overestimation therefore appears limited. Further research aimed at identifying potential red flags during ethics review may support RECs in detecting implausible effect size assumptions, thereby potentially helping to prevent underpowered studies and reduce research waste.

## Supplementary Information

Supplementary Material 1.

## Data Availability

The raw data used in this study is confidential and cannot be shared publicly. Access may be granted under specific circumstances and will require approval from METC LDD.

## Abbreviations

CCMO: Central Committee on Research Involving Human Subjects
CI: Confidence interval
CONSORT: Consolidated Standards of Reporting Trials
CTIS: Clinical Trials Information System
ICH: International Council on Harmonisation of Technical Requirements for Registration of Pharmaceuticals for Human Use
IQR: Interquartile range
REC: Research ethics committee
SPIRIT: Standard Protocol Items: Recommendations for Interventional Trials
