# Supplementary material for: The role of research ethics committees in addressing optimism in sample size calculations: a meta-epidemiological study
Source: Res Integr Peer Rev. 2025 Dec 12;10:26. doi: 10.1186/s41073-025-00184-w (PMC12699925; doi:10.1186/s41073-025-00184-w)
Supplement: Supplementary file 1 — Supplementary Material 1. [file 41073_2025_184_MOESM1_ESM.docx]

**Supplementary Material for**

**The role of research ethics committees in addressing optimism in sample size calculations: a meta-epidemiological study**

Marieke S. Jansen ^1^ ; Rolf H.H. Groenwold ^1 2^ ; Olaf M. Dekkers ^1 3 4^

^1^ Department of Clinical Epidemiology, Leiden University Medical Centre, Leiden, the Netherlands
^2^ Department of Biomedical Data Sciences, Leiden University Medical Centre, Leiden, the Netherlands
^3^ Department of Endocrinology and Metabolic Disorders, Leiden University Medical Centre, Leiden, the Netherlands
^4^ Department of Clinical Epidemiology, Aarhus University and Aarhus University Hospital, Aarhus, Denmark

Contents

[***Background information and structure REC (2015–2018)*** 2](#_Toc210977987)

[***Calculation of standardised effect sizes*** 3](#_Toc210977988)

[***List of trial registration numbers of included trials*** 5](#_Toc210977989)

[***Additional analyses*** 7](#_Toc210977990)

[1. Stratification by sample size comments 7](#_Toc210977991)

[**Table S1**. Completeness of reporting in trials without any sample size comments issued by REC 7](#_Toc210977992)

[2. Stratification by significance of trial results 8](#_Toc210977993)

[**Table S2**. Completeness of reporting in negative and positive trials 8](#_Toc210977994)

# ***Background information and structure REC (2015–2018)***

The Research Ethics Committee (REC) under study was the Medical Research Ethics Committee Leiden The Hague Delft (METC LDD), located at Leiden University Medical Centre (LUMC), the Netherlands. During the study period (2015–2018) the committee operated as the *Commissie Medische Ethiek (CME)*; in 2019 it merged with METC Zuid-West Holland (ZWH) to form the current METC LDD.

Structure in 2015-2018

- **Composition:** 16–26 members, including two biostatisticians.
- **Workload:** 122–145 protocols reviewed annually (60–70 interventional trials, remainder observational), approximately 3 per week.
- **Review process:** Each protocol was evaluated by members in relevant roles (e.g., physician, paediatrician, jurist, patient representative, pharmacist, medical device expert, methodologist, ethicist). A methodologist (biostatistician) was consistently assigned to each protocol, and assessed study design, endpoints, sample size, populations, timelines, and analysis plan.
- **Guidance for investigators:** In the Netherlands, the Central Committee on Research Involving Human Subjects (CCMO) provides a standardised protocol template with brief instructions for investigators, along with general submission guidance on its website. The website of METC LDD outlines procedures and regulations but, like the CCMO, does not routinely provide methodological support (e.g., for sample size determination). While investigators could consult METC LDD or the statistical department for specific questions, the responsibility to seek such support lay with the investigators themselves.

# ***Calculation of standardised effect sizes***

Standardised target effect sizes were calculated for the first submitted protocol and any revised versions if sample size calculations changed during review or amendments, using **Formula 1.1**. If the sample size was adjusted for attrition, **Formula 1.2** was used to retrieve the unadjusted sample size. Standardised observed effect sizes were calculated with **Formula 1.3** using the observed p-value. If a truncated p-value was reported (e.g., p < 0.05, p < 0.01, etc.), **Formulae 1.4-1.5** were used to calculate the Z-statistic and estimate the corresponding standardised observed effect size.

**Formula 1.1** Standardised target effect size

$$\delta=\frac{\sqrt{(r+1)}(Z_{1-\beta}+Z_{1-\alpha/2})}{\sqrt{r}*\sqrt{n_{A}}}$$

$\delta$ = standardised target effect size

$n_{A}$ = sample size (smallest arm)

$\alpha$ = type I error probability (i.e., false positive rate; probability to reject $H_{0}$ while $H_{0}$ is true; significance level)

$\beta$ = type II error probability (i.e., false negative rate; probability to not reject $H_{0}$ while $H_{0}$ is false)

$1-\beta$ = power (true positive rate; probability to reject $H_{0}$ while $H_{1}$ is true)

$Z_{1-\beta}$ = corresponding Z-value of cumulative probability of $1-\beta$

$Z_{1-\alpha/2}$ = corresponding Z-value of cumulative probability $1-\alpha/2$

$r$ = allocation ratio

**Formula 1.2** Retrieving original sample size after attrition correction

$$n=\left( 1-A \right)*n_{adj}$$

$n$ = sample size
$n_{adj}$ = sample size adjusted for attrition
$A$ = attrition rate (i.e., probability of participants to drop out of the study)

**Formula 1.3** Standardised observed effect size (using p-value)

$$\delta_{observed}=\varphi^{-1}\left( pvalue \right)* \sqrt{\frac{1}{n_{A}}+\frac{1}{n_{B}}}$$

$\varphi^{-1}\left( pvalue \right)$ = corresponding Z-value of the observed p-value
$n_{A}$ = target sample size of arm A
$n_{B}$ = target sample size of arm B

**Formula 1.4** Standardised observed effect size (using Z-statistic)

$$\delta_{observed}=Z* \sqrt{\frac{1}{n_{A}}+\frac{1}{n_{B}}}$$

$Z$ = Z-statistic
$n_{A}$ = target sample size of arm A
$n_{B}$ = target sample size of arm B

**Formula 1.5** Calculating the Z-statistic

$$Z=\frac{d}{SE(d)}$$

$$Z=\frac{ln[OR]}{SE(ln[OR])}$$

$$Z=\frac{ln[RR]}{SE(ln[RR])}$$

$$Z=\frac{ln[HR]}{SE(ln[HR])}$$

$d$ = observed difference. Applicable to effect size types: mean difference, difference in proportions, absolute risk reduction, regression coefficients, analysis of variance/covariance (ANOVA/ANCOVA) coefficients
$OR$ = Odds ratio
$RR$ = Risk ratio
$HR$ = Hazards ratio
$SE$ = Standard error

# ***List of trial registration numbers of included trials***

| **Dutch national registration number**  **www.onderzoekmetmensen.nl** | **Dutch trial register (old)**  **www.onderzoekmetmensen.nl** | **EudraCT number**  **www.clinicaltrialsregister.eu** | **NCT number**  **www.clinicaltrials.gov** |
| --- | --- | --- | --- |
| NL50201.058.14 | NTR4855 |  |  |
| NL51205.058.14 | NTR4853 | 2014-004472-35 |  |
| NL50646.058.14 |  | 2014-001389-93 | NCT02221869 |
| NL51585.058.14 |  |  |  |
| NL51850.058.14 |  |  | NCT02498106 |
| NL50627.058.15 |  | 2013-001178-20 |  |
| NL51904.058.15 |  | 2014-001096-31 | NCT02384538 |
| NL52829.058.15 | NTR5380 |  |  |
| NL52477.058.15 | NTR5263 | 2015-000687-33 |  |
| NL51603.058.15 |  | 2014-000148-14 | NCT02371369 |
| NL53076.058.15 |  | 2014-004932-20 | NCT02443298 |
| NL53528.058.15 |  | 2014-005112-42 | NCT02453061 |
| NL53159.058.15 |  |  | NCT02378844 |
| NL53850.058.15 |  | 2015-002488-40 | NCT02495662 |
| NL50426.058.15 |  | 2014-000363-40 | NCT02099747 |
| NL54235.058.15 |  | 2015-001098-42 | NCT02553317 |
| NL53081.058.15 |  | 2013-003413-18 |  |
| NL50844.058.15 |  |  |  |
| NL54040.058.15 |  | 2015-001275-50 | NCT02504268 |
| NL55837.058.15 |  | 2015-005258-37 |  |
| NL55839.058.15 |  | 2015-005259-28 |  |
| NL55586.058.15 |  |  |  |
| NL56370.058.15 |  |  |  |
| NL54882.058.15 |  |  | NCT01479283 |
| NL57170.058.16 |  | 2016-000852-91 | NCT02499237 |
| NL57263.058.16 |  |  |  |
| NL56487.058.16 |  | 2012-002535-28 | ISRCTN97443826 |
| NL58537.058.16 |  | 2013-001506-29 | NCT02611687 |
| NL57115.058.16 |  | 2016-001038-91 | NCT02900443 |
| NL58933.058.16 |  | 2016-000602-10 | NCT02814019 |
| NL58509.058.16 |  | 2016-002211-18 | NCT03096834 |
| NL58595.058.16 |  | 2016-000401-36 | NCT03373968 |
| NL60821.058.17 | NL7844 |  |  |
| NL60858.058.17 |  | 2017-000583-15 | NCT03104426 |
| NL62050.058.17 | NTR7295; NL7097 |  |  |
| NL60839.058.17 |  |  | NCT03212092 |
| NL62540.058.17 |  |  |  |
| NL61526.058.17 |  | 2017-000737-31 | NCT03252353 |
| NL62897.058.17 | NTR6878; NL6708 |  |  |
| NL61964.058.17 |  |  | NCT03266185 |
| NL61255.058.17 |  | 2015-003153-18 | NCT03070392 |
| NL63735.058.17 |  |  | NCT03588104 |
| NL62767.058.17 | NTR7175 |  | NCT03611517 |
| NL64330.058.17 | NTR7516; NL7284 |  |  |
| NL63892.058.18 |  |  | NCT03811587 |
| NL64347.058.17 |  | 2017-002446-76 | NCT03353753 |
| NL65658.058.18 | NTR7581 |  |  |
| NL65236.058.18 | NTR7376 |  |  |
| NL65719.058.18 | NL7365 |  |  |
| NL66018.058.18 |  | 2017-001629-41 | NCT03238235 |

# ***Additional analyses***

## Stratification by sample size comments

Of the 50 protocols included, 41 (82%) had no comments regarding the sample size calculation. Among these, 34 trials (83%) appeared to overestimate their target effect size. Across all trials without any sample size comments, the median overestimation was 0.22 [IQR: 0.04–0.41].

### Table S1. Completeness of reporting in trials without any sample size comments issued by REC

|  |  | Final approved protocol ^b^ | |
| --- | --- | --- | --- |
|  |  | n = 41 | % |
| Complete | | 20 | 49 |
| Calculation absent | | 0 | 0 |
| Missing parameters | |  |  |
|  | Type I error (alpha) | 2 | 5 |
|  | Type II error (beta) | 0 | 0 |
|  | One - or two-sided hypothesis test | 15 | 37 |
|  | Target effect size | 0 | 0 |
|  | Variability or event rate control arm | 4 | 10 |
|  | Other ^a^ | 3 | 7 |
| Target effect size not substantiated | | 21 | 51 |
| Variability not substantiated | | 12 | 29 |

^a^ E.g., correlation between repeated measurements if applicable, number of required events in time-to-event analyses, number of required participants
^b^ Most recently approved protocol version (e.g., either after initial ethics review, or after amendments if applicable)

Details regarding comments on justification of the target effect size

REC comments on the justification of the target effect size were issued in only 3 cases (6%), with target effect sizes of 0.70, 0.83, and 1.06. Notably, two of these had some form of justification, and one had none. Among the 47 protocols without such comments, 30 (64%) had a target effect size of 0.5 or larger, and 9 (19%) even 0.8 or larger; yet justification was missing in 43% (13/30) and 22% (2/9) of these, respectively.

## Stratification by significance of trial results

As shown in **Figure 2** of the main manuscript, the overestimation of the target effect size was inherently more pronounced in trials with negative results. All negative trials showed an overestimation of the target effect size, with a median overestimation of 0.41 [IQR: 0.33–0.49].

Negative trials were more frequently observed among investigator-initiated trials (57%, 20/35) compared to industry-sponsored trials (33%, 5/15). Interestingly, the distribution of target effect sizes was not necessarily larger in magnitude in negative trials (median 0.55 [IQR: 0.46–0.72]) compared with positive trials (median 0.66 [IQR: 0.51–0.75]).

### Table S2. Completeness of reporting in negative and positive trials

|  |  | Final approved protocol ^b^ | | | |
| --- | --- | --- | --- | --- | --- |
|  |  | Negative trials | | Positive trials | |
|  |  | n = 25 | % | n = 25 | % |
| Complete | | 12 | 48 | 13 | 52 |
| Calculation absent | | 0 | 0 | 0 | 0 |
| Missing parameters | |  |  |  |  |
|  | Type I error (alpha) | 1 | 4 | 2 | 8 |
|  | Type II error (beta) | 0 | 0 | 0 | 0 |
|  | One - or two-sided hypothesis test | 11 | 44 | 9 | 36 |
|  | Target effect size | 0 | 0 | 0 | 0 |
|  | Variability or event rate control arm | 6 | 24 | 3 | 12 |
|  | Other ^a^ | 2 | 8 | 2 | 8 |
| Target effect size not substantiated | | 13 | 52 | 10 | 40 |
| Variability not substantiated | | 5 | 20 | 9 | 36 |

^a^ E.g., correlation between repeated measurements if applicable, number of required events in time-to-event analyses, number of required participants
^b^ Most recently approved protocol version (e.g., either after initial ethics review, or after amendments if applicable)
